# Reliability and validity of the Japanese translation of the Eating Disorders Quality of Life (ED-QOL) scale for Japanese healthy female university undergraduate students and patients with eating disorders

**DOI:** 10.1186/s13030-020-00189-5

**Published:** 2020-07-31

**Authors:** Ryo Yoneda, Makoto Otani, Maiko Hiraide, Takeshi Horie, Tomoyo Mitsui, Toshiyuki Yoshida, Gen Komaki, Kazuhiro Yoshiuchi

**Affiliations:** 1 Department of Psychosomatic Medicine, The University of Tokyo Hospital, 7-3-1 Hongo, Bunkyo-ku, Tokyo, 113-8655 Japan; 2 Department of Psychology, Faculty of Human Development and Education, Kobe Shinwa Women’s University, 7-13-1 Suzurandaikitamachi Kita-Ku, Kobe, Hyogo 651-1111 Japan; 3 School of Health Sciences at Fukuoka, International University of Health and Welfare, 137-1 Enokizu, Okawa, Fukuoka, 831-8501 Japan; 4 Department of Stress Sciences and Psychosomatic Medicine, Graduate School of Medicine, The University of Tokyo, 7-3-1 Hongo, Bunkyo-ku, Tokyo, 113-8655 Japan

**Keywords:** Eating disorders quality of life (ED-QOL), Reliability, Validity

## Abstract

**Background:**

The Eating Disorder Quality of Life (ED-QOL) scale is a 25-item self-report measure that assesses health-related quality of life (HRQoL) of eating-disorder patients. Although the ED-QOL is one of the most widely used questionnaires in many countries, no prior research has addressed the psychometric properties of the Japanese translation of the ED-QOL. Therefore, the aim of the present study was to assess its reliability and validity.

**Methods:**

A total of 99 Japanese female eating disorder patients and 469 female healthy university undergraduate students completed the Japanese translation of the ED-QOL in addition to the Eating Attitudes Test-26 (EAT-26) and Eating Disorder Inventory-2 (EDI-2). The patient group consisted of 37 patients with anorexia nervosa restricting type (AN-R), 35 patients with binge-eating/purge type (AN-BP), and 27 patients with bulimia nervosa (BN). We performed confirmatory factor analyses on the ED-QOL subscales both for Japanese eating disorder patients and for healthy university undergraduate students. Reliability was assessed using internal consistency indicated by Cronbach alpha coefficients and convergent validity was assessed using Pearson’s correlation coefficients. To assess group differences between the eating disorder patients and healthy university undergraduate students, Student’s t-tests were conducted.

**Results:**

The CFA showed that the CFI was .90 and RMSEA was .084 (90% confidence interval = .079–.088). The internal consistency of the ED-QOL varied from good to excellent. The EAT-26 total score and three subscales and the EDI-2 subscales had significant correlations with the ED-QOL global QOL score and four subscales. There were no significant correlations between the EDI-2 subscale “Body Dissatisfaction” and the ED-QOL subscales “Physical/Cognitive” and “Work/School”. Eating disorder patients scored significantly higher than healthy university undergraduate students on all ED-QOL subscales and the global QOL score.

**Conclusions:**

Based on this study, the Japanese translation of the ED-QOL can be regarded as reliable, valid, and functional for female eating-disorder patients and female healthy university undergraduate students.

## Background

Eating-disorder patients have serious physical, psychological, social, and role functioning difficulties, which are central to the concept of health-related quality of life (HRQoL) [1]. As such, HRQoL is one of the most important end-points in the treatment of enduring eating disorders [2]. The Eating Disorder Quality of Life scale (ED-QOL) [1], which contains disease-specific HRQoL measures, is more sensitive than a generic measure for identifying meaningful differences by diagnosis [3]. Most of the other existing questionnaires focus on eating behaviors and cognitive aspects of eating disorders, but ED-QOL evaluates health-related QOL, including social aspects, so we can assess the therapeutic effects by evaluating social functioning and its change. Measuring changes in patients’ clinical presentations when symptoms show minimal improvement may be valuable for motivating patients toward behavior change [3].

ED-QOL is a 25-item self-report measure assessing the HRQoL of eating-disorder patients. The scale includes four subscales (Psychological, Physical/Cognitive, Financial, and Work/School), which can be combined into a global QOL score. The adequacy of this structure was previously checked and considered acceptable in German translations of the ED-QOL [4]. The ED-QOL has shown excellent psychometric properties including adequate reliability and validity [1].

The ED-QOL has been used in prior studies to evaluate the HRQoL of eating disorder patients [2, 3, 5]. Although the ED-QOL is one of the most widely used questionnaires for assessing the HRQoL of eating-disorder patients [4, 6, 7], no research has addressed the psychometric properties of a Japanese translation of the ED-QOL. Therefore, the aim of the present study was to assess the reliability and validity of the Japanese translation of the ED-QOL. This can be used for endpoints of epidemiological surveys and of intervention studies in Japan, as well as for cross-country comparisons.

## Methods

### Participants and procedure

We collected patient data at The University of Tokyo Hospital. The healthy control group data was collected by universities in Japan for another study by Mitsui [8]. The sample consisted of 99 Japanese female eating disorder patients, diagnosed according to DSM-5 by experienced clinicians, and 469 healthy female undergraduate students. The eating disorder patients consisted of 37 anorexia nervosa restricting type (AN-R) patients, 35 anorexia nervosa binge-eating/purge type (AN-BP) patients, and 27 bulimia nervosa (BN) patients. All participants completed the ED-QOL, Eating Attitudes Test-26 (EAT-26) Eating Disorder Inventory-2 (EDI-2), and a questionnaire about the frequency of binge-eating, vomiting, laxative use, and compulsive exercise.

### Measures

#### Japanese translation of the eating disorders quality of life

The original translation of the ED-QOL has been shown to have high overall internal consistency and high discriminant and convergent validity [1]. Participants responded to items on a scale from 0 (never) to 4 (always). Scaled scores are item scores which are calculated by averaging the items of each subscale. The total score is calculated by averaging the scores of all of the items of the EDQOL. Higher scores indicate worse ED-QOL.

The translation of the ED-QOL into Japanese was carefully done by one of the authors (GK) with the permission by the corresponding author of the original version to ensure that there were no differences in nuance between the original English and the new Japanese translation. The Japanese translation was back-translated into English by a professional native editor who was fluent in both English and Japanese. The back-translated ED-QOL was then confirmed for accuracy by the authors of the original English version.

#### Eating attitudes Test-26

The Eating Attitudes Test-26 (EAT-26) is a 26-item, 6-point scale (1 (never) to 6 (always)), self-report measure. A total score of 20 or higher is highly correlated with a diagnosis of anorexia nervosa or bulimia [9]. The EAT-26 has been validated both in clinical populations and in nonclinical adult and adolescent people [10]. The Japanese version of the EAT-26 has also been favorably evaluated for reliability and validity [11, 12]. In this study, we examined the total score and three subscales: Dieting, Bulimia and Food Preoccupation, and Oral control.

#### Eating disorder Inventory-2

In 1991, Garner added 27 items and three subscales to the Eating Disorder Inventory (EDI) in order to develop the EDI-2 [13], which is a 91-item, 6-point scale (i.e. “always,” “usually,” “often,” “sometimes,” “rarely,” or “never”), self-report measure. The EDI-2 has been favorably evaluated for reliability and validity in other countries, and a Japanese version of the EDI-2 was also developed and validated [14]. The EDI-2 items form 11 subscales, and similar to a prior study, we examined three of these subscales: Drive for Thinness (DT), Body Dissatisfaction (BD) and Bulimia (B). We specifically chose these three subscales because they represented the core symptoms pertaining to the diagnostic criteria of eating disorders.

### Ethical considerations

The protocol was approved by the Ethics Committee of the Graduate School of Medicine, The University of Tokyo [10800], the National Center of Neurology and Psychiatry [A2011–090], and the International University of Health and Welfare [13-Io-180], and written informed consent was provided by all subjects prior to enrollment in the study.

### Statistical analysis

We performed confirmatory factor analyses (CFA) on the ED-QOL subscales for participants. The adequacy of the models was evaluated using the comparative fit index (CFI), and the Root Mean Square Error of Approximation (RMSEA). The reliability of the ED-QOL was assessed by means of its internal consistency using Cronbach alpha coefficients for both the individual ED-QOL subscales as well as for the total score. Concurrent validity for the EAT-26 and EDI-2 was assessed by means of Pearson’s correlation coefficients. To assess group differences, an ANOVA and a Scheffe F test was performed between eating disorder patients and healthy university undergraduate students. Significance levels were set at *p* < 0.05. All analyses were performed using the IBM SPSS version 22 and the IBM SPSS Amos™ 22 J.

## Results

The characteristics of the participants are summarized in Table 1.

**Table 1:** Participant characteristics

	Eating disorder patients	healthy university undergraduate students (*n* = 469)
	(*n* = 99)	
age(years)	32.8 ± 12.1	19.9 ± 1.7
body mass index(kg/m2)	18.3 ± 5.8	20.7 ± 2.8
occupation		
student	23	469
full-time worker	16	0
part-time worker	18	0
homemaker/unemployed/on leave	42	0
Binge-eating (per 4 weeks)	8.9 ± 22.4	1.7 ± 3.7
Vomiting (per 4 weeks)	8.3 ± 18.3	0.1 ± 0.8
Use of laxatives (per 4 weeks)	2.7 ± 8.7	0.1 ± 1.6
Compulsive exercise (per 4 weeks)	3.9 ± 8.4	0.4 ± 1.5
EAT-26		
total score	24.5 ± 16.1	8.7 ± 6.2
Dieting	13.1 ± 9.1	6.2 ± 4.2
Bulimia and Food Preoccupation	5.7 ± 5.3	1.0 ± 1.7
Oral Control	5.8 ± 5.1	1.5 ± 2.3
EDI-2		
Drive for Thinness (DT)	9.1 ± 6.6	5.6 ± 4.9
Body Dissatisfaction (BD)	15.3 ± 7.4	16.2 ± 7.1
Bulimia (B)	5.7 ± 6.6	2.6 ± 3.5

*EDI-2* Eating Disorder Inventry-2, *ED-QOL* Eating Disorder Quality of Life

### Confirmatory factor analyses (CFA)

**Fig. 1:**
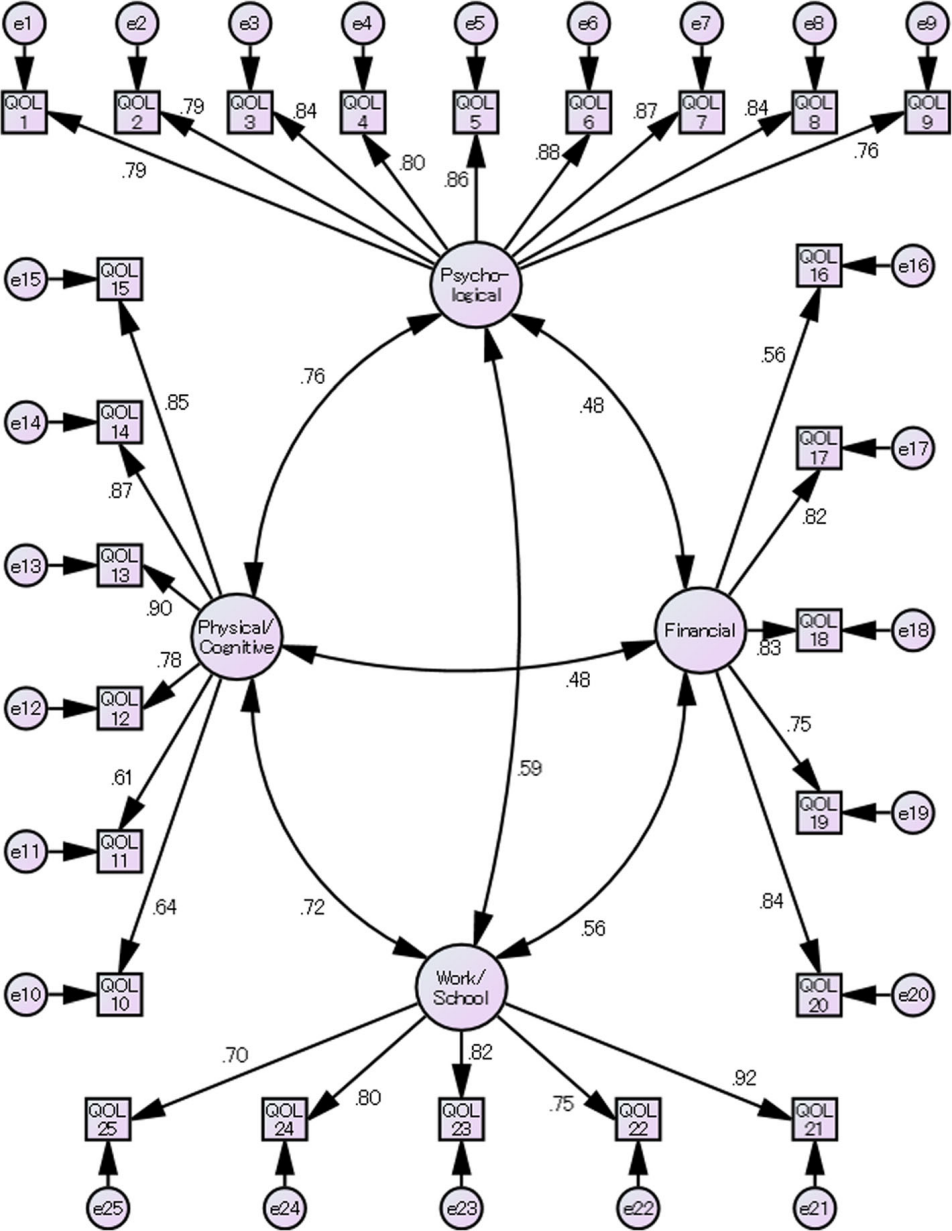
Path diagram for the confirmatory factor analysis. All of the numbers show the standard partial regression coefficients and correlation coefficients and all of the paths are statistically significant with *P* value smaller than 0.001. Psychological, Physical/Cognitive, Financial and Work/School represent subscales of the Eating Disorders Quality of Life. QOL1–25 represents question numbers 1–25 of the Eating Disorders Quality of Life. e1–25 are all residual errors of the model

We performed a CFA to check the fit of the 4-factor structure (i.e. Psychological, Physical/Cognitive, Financial, and Work/School) as proposed in the original ED-QOL. The CFA showed that the CFI was .90, which is considered indicative of a good fit [15], and that the RMSEA was .084 (90% confidence interval = .079–.088), which is considered acceptable [16]. We named these four subscales Psychological (nine items), Physical/Cognitive (six items), Financial (five items), and Work/School (five items) following the original ED-QOL. Figure 1 shows the path diagram. All standard partial regression coefficients and correlation coefficients are statistically significant.

### Internal consistency

Table 2 presents the internal consistency results for the four subscales and the global QOL score. The internal consistency of the ED-QOL varied from 0.84 to 0.95.

**Table 2:** Internal consistency (Cronbach alpha) of the Japanese translation of the ED-QOL subscales and global QOL scores (*n* = 539–561)^a^

Subscales	Cronbach alpha
Psychological	0.95
Physical/Cognitive	0.89
Financial	0.84
Work/School	0.89
global QOL score	0.95

^a^Sample sizes vary per subscale because not all participants answered all ED-QOL items

### Correlation with EAT-26 and EDI-2

In an initial attempt to explore the validity of the Japanese translation of the ED-QOL, we examined the Pearson’s correlation coefficients between the ED-QOL scores and those of the other two questionnaires (Table 3).

**Table 3:** Pearson’s correlation coefficients between the EAT-26, EDI-2, and ED-QOL

	ED-QOL Psychological	Physical/Cognitive	Financial	Work/School	global QOL score
**EAT-26**					
total score	0.71***	0.65***	0.45***	0.55***	0.74***
Dieting	0.68***	0.52***	0.41***	0.42***	0.65***
Bulimia and Food Preoccupation	0.67***	0.64***	0.51***	0.55***	0.73***
Oral control	0.39***	0.63***	0.20***	0.47***	0.47***
**EDI-2**					
DT	0.66***	0.40***	0.32***	0.28***	0.58***
BD	0.34***	0.06	0.16***	0.02	0.23***
B	0.63***	0.45***	0.49***	0.35***	0.61***

*p < .05, **p < .01, ***p < .001

*DT* Drive for Thinness, *BD* Body Dissatisfaction, *B* Bulimia, *n.s.* not significant

The EAT-26 total score and those of all three subscales had significant correlations with the ED-QOL global score and its four subscales. There were no significant correlations between the EDI-2 “Body Dissatisfaction” subscale and the ED-QOL “Physical/Cognitive” and “Work/School” subscales.

### Group differences

In all subscale scores and the global QOL score, ANOVA results determined that there were statistically significant differences between group means. In post-hoc analysis, eating disorder patients had significantly higher scores than healthy university undergraduate students, with the exception of AN-R subtype participants in the financial subscale score (Table 4).

**Table 4:** Mean and SD of the ED-QOL subscales and global QOL score

	eating disorder patients			healthy university undergraduate students (*n* = 469)d	ANOVA	group difference (*p* < .01)†
	AN-R (*n* = 35–37)a	AN-BP (*n* = 33–34)b	BN (*n* = 25–27)c			
Psychological	1.32 ± 1.06	2.36 ± 1.20	2.62 ± 0.85	0.70 ± 0.82	F(3, 564) = 81.3, *p* < .001	b,c > a > d
Physical/Cognitive	1.03 ± 0,87	1.90 ± 1.24	1.74 ± 1.13	0.33 ± 0.56	F(3, 563) = 98.6, p < .001	b, c > a > d
Financial	0.05 ± 0.03	0.65 ± 0.98	1.14 ± 1.26	0.11 ± 0.33	F(3, 563) = 53.6, p < .001	b > c > a, d
Work/School	0.47 ± 0.87	1.27 ± 1.17	1.01 ± 1.10	0.07 ± 0.27	F(3, 559) = 93.7, p < .001	b, c > a > d
global QOL score	0.80 ± 0.60	1.70 ± 0.89	1.85 ± 0.92	0.37 ± 0.45	F(3, 564) = 133.5, p < .001	b, c > a > d

†Results of the Scheffe F test

## Discussion

This study aimed to assess the reliability and validity of the Japanese translation of the ED-QOL. Cronbach alpha coefficients of the global score and those of the four subscales in the present study were comparable with those observed in a previous study (.86 for the Physical/Cognitive subscale, .95 for the Psychological subscale, .84 for the Work/School subscale, .86 for the Financial subscale, and .94 for the overall alpha coefficient) [1]. The Japanese translation of the ED-QOL showed good-to-excellent internal consistency in all ED-QOL subscales and in the global score. The CFA in the current study also demonstrated acceptable fit indices, showing that the models were adequate, and that the four ED-QOL subscales are acceptable.

The concurrent validity of the ED-QOL was evaluated by examining whether or not the subscale scores correlated with the EAT-26 and the EDI-2. The four ED-QOL subscales and global score showed moderate correlations with most of the EAT-26 and the EDI-2 subscales.

Regarding group differences, in the global scores and all subscale scores, eating disorder patients had significantly higher scores on the ED-QOL than healthy university undergraduate students, as similarly indicated by a previous study [3]. These results showed that, using these scores, we may be able to distinguish between potential eating disorder patients and healthy university undergraduate students. These results supported the construct validity of the ED-QOL global score and all subscale scores. In addition, there were no significant differences between AN-BP and BN participants on the ED-QOL except on the Financial subscale. There were significant differences in scores on the Financial subscale of the ED-QOL among the AN-R, AN-BP and BN groups. This may be due to the fact that BN patients were similar to AN-BP patients on the HRQoL, but BN patients spent more money on binge eating than AN-BP patients. In contrast, there was no significant difference between AN-R patients and healthy university undergraduate students on the Financial subscale. This may be because AN-R patients, who did not display symptoms of binge-eating, hardly ever had financial problems with the disorder. These differences were more significant than indicated by a previous study [3], potentially due to the fact that most of the eating disorder patients of the previous study were classified as AN-R (85 of 131 were AN-R except Eating Disorder, Not Otherwise Specified).

The values of the ED-QOL global score and its four subscales in the present study were also comparable with those observed in a previous study (Psychological 2.20 ± 0.96, Physical/Cognitive 1.52 ± 0.92, Financial 0.49 ± 0.80, Work/School 0.29 ± 0.62, Total 1.33 ± 0.67) [1]. Even when divided by eating disorder subtype, the values of the ED-QOL global score and the four subscales in the present study were comparable with those observed in a previous study (AN-R: Psychological 1.8 ± 1.1, Physical/Cognitive 1.1 ± 1.0, Financial 0.3 ± 0.7, Work/School 0.3 ± 0.6; AN-BP: Psychological 2.4 ± 0.8, Physical/Cognitive 1.8 ± 0.9, Financial 1.2 ± 1.1, Work/School 1.0 ± 1.0; BN: Psychological 1.7 ± 1.0, Physical/Cognitive 1.0 ± 1.1, Financial 0.3 ± 0.6, Work/School 0.3 ± 0.6) [3]. These scores did not significantly differ. There are some limitations to this study. First, there was a large age difference between the eating-disorder patients and healthy university undergraduate students, which may be related to some of the differences in result (e.g. “Financial” and “Work/School”). Second, since this study was cross-sectional, it is unclear as to if changes over time can be detected without a follow up. Third, in the present study, all eating-disorder patients were female, however, this is consistent with the previous study method of the original version of the ED-QOL [1]. Further studies are needed to assess the use of the ED-QOL with male eating-disorder patients.

## Conclusion

The results from this study indicate that the Japanese translation of the ED-QOL is reliable and valid for female eating-disorder patients and female healthy university undergraduate students. Therefore, the Japanese translation of the ED-QOL is useful in the assessment of the HRQoL of Japanese eating disorder patients. In clinical use, we can assess the therapeutic effects by evaluating social functioning and its change in Japanese patients, which may be valuable for motivating Japanese patients toward behavior change.

## Data Availability

The datasets used or analyzed during the current study are available from the corresponding author on reasonable request.

## Abbreviations

ED-QOL: Eating Disorder Quality of Life
EAT-26: Eating Attitudes Test-26
EDI-2: Eating Disorder Inventory-2
AN-R: Anorexia nervosa restricting type
AN-BP: Anorexia nervosa binge-eating/purge type
BN: Bulimia nervosa
EDI: Eating Disorder Inventory
DT: Drive for Thinness
BD: Body Dissatisfaction
B: Bulimia
AN: Anorexia nervosa
DSM-5: Diagnostic and Statistical Manual of Mental Disorders, Fifth Edition
CFA: Confirmatory factor analysis
HRQOL: Health-related quality of life

## References

[1] Engel SG, Wittrock DA, Crosby RD, Wonderlich SA, Mitchell JE, Kolotkin RL (2006). Development and psychometric validation of an eating disorder-specific health-related quality of life instrument. Int J Eat Disord.

[2] Bamford B, Barras C, Sly R, Stiles-Shields C, Touyz S, Le Grange D, Hay P, Crosby R, Lacey H (2015). Eating disorder symptoms and quality of life: where should clinicians place their focus in severe and enduring anorexia nervosa?. Int J Eat Disord..

[3] Ackard DM, Richter S, Egan A, Engel S, Cronemeyer CL (2014). The meaning of (quality of) life in patients with eating disorders: a comparison of generic and disease-specific measures across diagnosis and outcome. Int J Eat Disord..

[4] Tagay S, Schlegl S, Senf W (2011). Validation of the German translation of the eating disorders quality of life (EDQOL). Psychother Psych Med.

[5] Mitchison D, Hay P, Engel S, Crosby R, Le Grange D, Lacey H, Mond J, Slewa-Younan S, Touyz S (2013). Assessment of quality of life in people with severe and enduring anorexia nervosa: a comparison of generic and specific instruments. BMC Psychiatry.

[6] Winkler LA, Hemmingsen SD, Gudex C, Blegvad AC, Støving RK, Arnfred SMH (2019). A Danish translation of the eating disorder quality of life scale (EDQLS). J Eat Disord.

[7] Lal M, Abraham S (2011). Translation of the quality of life for eating disorders questionnaire into Hindi. Eat Behav.

[8] Mitsui T, Yoshida T, Komaki G (2017). Psychometric properties of the eating disorder examination questionnaire in Japanese adoelscents. Biopsychosoc Med.

[9] Garner DM, Garfinkel PE (1979). The eating attitudes test: an index of the symptoms of anorexia ervosa. Psychol Med.

[10] Williams RL, Shaefer CA, Shisslak CM, Gronwold VH, Comercio GD (1986). Eating attitudes and behaviors in adolescent women: discrimination of normal, dieters, and suspected bulimics using the eating attitudes test and eating disorder inventory. Int J Eat Disord..

[11] Nakai Y (2003). Validity of the Japanese version of eating attitudes test (EAT). Seishin Igaku.

[12] Mukai T, Crago M, Shisslak CM (1994). Eating attitudes and weight preoccupation among female high school students in Japan. J Child Psychol Psychiatry.

[13] Garner DM (1991). Eating disorder Inventory-2: professional manual.

[14] Ando T, Ichimaru Y, Konjiki F, Shoji M, Komaki G (2007). Variations in the preproghrelin gene correlate with higher body mass index, fat mass, and body dissatisfaction in young Japanese women. Am J Clin Nutr.

[15] Hoyle RH, Hoyle RH (1995). The structural equation modeling approach: basic concepts and fundamental issues. Structural equation modeling, concepts, issues, and applications.

[16] Browne MW, Cudeck R, Bollen KA, Long JS (1993). Alternative ways of assessing model fit. Testing structural equation models.

